# OA08.02. Differential acute effects of real and sham acupuncture on mu-opioid receptor availability in treatment naive and conditioned chronic pain patients

**DOI:** 10.1186/1472-6882-12-S1-O30

**Published:** 2012-06-12

**Authors:** N Akbar, J Zubieta, T Love, D Clauw, R Harris

**Affiliations:** 1 University of Michigan, Ann Arbor, USA

## Purpose

Previous studies suggest acupuncture analgesic effects involve the release of endogenous opioid peptides. We have previously shown that while both acupuncture and sham reduced clinical pain in fibromyalgia (FM), only acupuncture increased mu-opioid receptor (MOR) baseline receptor availability *in vivo* (binding potential, BP). Here we investigated whether real and sham acupuncture were associated with differential changes in MOR BP in FM patients that were naive to acupuncture as compared to conditioned patients.

## Methods

20 female FM patients (mean (SD) age 44.3 (13.6) years) were randomized to acupuncture (n=10) or sham (n=10) treatments over four weeks. Acupuncture involved insertion of nine needles into the body whereas sham did not penetrate the skin. Positron emission tomography (PET) with the mu-opioid radiotracer [11C] carfentanil was performed during the first and ninth treatments. Each session included a ‘baseline’ scan prior to needle insertion and ‘treatment’ scan during needle insertion. We compared the difference in MOR BP between baseline and treatment, before and after 4 weeks of acupuncture versus sham. MOR quantification performed with Logan plots resulting in voxel-by-voxel maps of MOR BP. Images were analyzed with SPM 5.

## Results

Real and sham acupuncture differed in their acute effects on MOR BP. The effect was localized in the left temporal pole and left amygdala/striatum (significant after correction for multiple comparisons, p<.05). The treatment effect was largely due to a change in BP with sham after 4 weeks.

## Conclusion

Acupuncture and sham resulted in differential acute effects on MOR binding in treatment naive as compared to conditioned patients. These findings could demonstrate a conditioned placebo response in sham treated chronic pain patients.

